# Knowledge, attitudes and practices of healthcare workers in paediatric HIV at Pelonomi Tertiary Hospital

**DOI:** 10.4102/sajhivmed.v26i1.1720

**Published:** 2025-08-30

**Authors:** Reatile Mabe, Michael A. Pienaar, Riana van Zyl

**Affiliations:** 1 Department of Paediatrics and Child Health, Faculty of Health Sciences, University of the Free State, Bloemfontein, South Africa

**Keywords:** knowledge, attitudes, practices, HIV, healthcare workers, paediatric, antiretroviral therapy, disclosure

## Abstract

**Background:**

Paediatric HIV remains a major public health challenge. Little is known about the HIV knowledge, perceptions, and behaviours of healthcare professionals caring for these children.

**Objectives:**

To assess the level of knowledge, attitudes and practices (KAP) of healthcare workers (HCW) caring for children with HIV in a tertiary hospital setting.

**Method:**

A cross-sectional study was conducted at Pelonomi Tertiary Hospital between July 2022 and September 2022. Healthcare workers, selected through purposive sampling, completed an anonymous self-administered KAP questionnaire on paediatric HIV.

**Results:**

There were 94 participants in this study; 62 were nurses and 32 were medical doctors. Less than half of the HCWs (44.7%) had adequate knowledge. Doctors (87.5%) were more knowledgeable than nurses (22.6%). Areas in which there was a significant difference in knowledge (*P* < 0.05) were in breastfeeding, vertical transmission prevention, management of HIV and tuberculosis co-infection, the use of the polymerase chain reaction test, and first-line treatment regimens. The attitudes of the majority of HCWs were favourable and optimistic. Regarding practices, more nurses (60.7%) wore gloves than doctors (37.5%). The majority of HCWs (85.7%) disposed of sharps appropriately.

**Conclusion:**

Despite the low levels of knowledge among study participants, particularly among the nursing group, favourable attitudes suggested that HCWs were willing to increase their levels of knowledge. Healthcare workers can be empowered in a supportive workplace by being offered interactive training sessions based on established guidelines.

**What this study adds:** This study demonstrates favourable healthcare worker attitudes but inadequate clinical knowledge in paediatric HIV care. It underscores the need for nurse-focused, guideline-based training and contributes current, locally relevant data to inform quality improvement and strengthen paediatric HIV service delivery in South Africa.

## Introduction

Paediatric HIV remains a significant global health challenge, with sub-Saharan Africa bearing the highest burden of disease. According to the WHO, an estimated 1.7 million children were living with HIV globally in 2023, with nearly 90% residing in sub-Saharan Africa.^1^ Specifically for South Africa, the estimated number of children (0–14 years) is estimated to be 160 000.^2^ South Africa’s progress for children living with HIV indicates that 81% of children with HIV have been diagnosed, 65% of those diagnosed are on antiretroviral therapy (ART), and 68% of those on ART have achieved viral suppression.^3,4,5,6^

Effective management in paediatric HIV is often complicated by issues such as tuberculosis co-infection, breastfeeding-related transmission risks, challenges in ART regimens, and timely diagnostic measures such as polymerase chain reaction (PCR) testing.

Children with HIV are 15–20 times more likely to develop active tuberculosis compared to their HIV-uninfected counterparts.^7^ A study from the Eastern Cape reported that 28.7% of HIV-infected children had prevalent tuberculosis at enrolment,^8^ while recent data from the Western Cape indicate persistently high tuberculosis incidence in children under 5 years with unsuppressed viral loads.^9^

Vertical transmission prevention (VTP) programmes – including maternal ART and infant prophylaxis – have been effective in significantly reducing HIV transmission rates during breastfeeding.^10^ Through the integration of HIV care into routine maternal and new-born services, vertical transmission of HIV has been reduced from nearly 30% in 2002 to less than 3% in 2022.^11^

Early diagnosis is vital for the early initiation of ART. This is associated with improved survival and developmental outcomes in HIV-infected infants.^9^ Polymerase chain reaction testing is the gold standard for diagnosing HIV in infants as early as 6 weeks of age. Delays in testing and linkage to care remain significant barriers to optimal paediatric HIV management.^12^

Since the inception of paediatric ART, there has been a shift towards more effective and child-friendly regimens.^13^ In South Africa, treatment is based on national guidelines as well as those set out by the WHO.^14,15^ National treatment guidelines for HIV in paediatrics have since been revised over the years, and this may contribute to healthcare worker (HCW) confusion.^16^

This study explored the knowledge, attitudes and practices (KAP) of HCWs involved in the care of HIV-infected paediatric patients in a tertiary hospital in the Free State, with particular emphasis on HIV and tuberculosis co-infection, breastfeeding practices, ART regimens, and testing. Doctors and nurses were used as the comparison groups for this KAP study as HIV in South Africa is managed by both doctors and Nurse Initiated Management of Antiretroviral Therapy (NIMART)-trained nurses. By examining these critical areas, the study aimed to identify gaps in practice and opportunities for strengthening paediatric HIV care within the South African context. The expectation is that both NIMART-trained nurses and doctors should have a similar level of knowledge with regard to testing algorithms, managing uncomplicated HIV, and initiating ART.

Even though paediatric HIV is of public health significance,^1^ there is limited evidence on the KAP of HCWs caring for HIV-infected paediatric patients. Knowledge, attitudes and practices studies are conducted by use of a survey that collects information representative of a specific target population.^17^ They aim to establish information on what is already known, what is believed, and what is done in practice. Structured or semi-structured questionnaires, either self-administered or administered by the researcher, can be used to collect both qualitative and quantitative data.^17^

In South Africa, KAP studies on HCWs caring for HIV-infected paediatric patients have mainly focused on barriers to providing services as well as HIV disclosure challenges in children but not much on paediatric care practices.^18,19^ A KAP study of HIV paediatric care was conducted in Cameroon in 2019,^16^ which found that children with HIV in Cameroon face challenges such as limited access to ART, inadequate healthcare resources, and insufficient training for HCWs.

Knowledge, attitudes and practice studies can serve as tools that identify barriers in healthcare delivery and facilitate targeted improvements in paediatric HIV care. An assessment of the KAP of HCW in our own setting is a good starting point for establishing the level of existing knowledge, with the aim to identify gaps and areas of improvement where needed.

## Research methods and design

### Study design

A descriptive cross-sectional study was conducted at Pelonomi Tertiary Hospital, South Africa.

### Setting

Participants were doctors and nurses working in the general paediatric wards, the paediatric intensive care unit, as well as the neonatal high care unit (including Kangaroo Mother Care and post-natal unit), in a tertiary hospital in Bloemfontein, Free State. Most admissions are referrals from local clinics, community health centres and district hospitals outside of the city.

### Study population and sampling strategy

Ninety-four participants were included in the study. Two categories of participants were asked to answer questions: (1) medical doctors, and (2) nursing personnel. The medical doctors consisted of first-year medical interns and paediatric registrars in various years of their training. The nursing personnel consisted of enrolled nurses and professional nurses.

Purposive sampling was used to enrol the study participants. The researcher personally addressed the participants, either individually or as a group (i.e. in meetings or lectures) before data collection. Approval to conduct the study was granted by both the Health Science Research Ethics Committee (HSREC) of a public university in the Free State and the provincial Department of Health.

### Data collection

Data collection took place from July 2022 to September 2022 using a paper-based, self-administered, and anonymous questionnaire. The tool was piloted on five individuals, who were subsequently included in the final study sample. Participant information included level of education, occupation, employment duration, and unit of occupation.

The questionnaire consisted of three sections addressing KAPs:

- Knowledge: Refers to the participant’s factual understanding of paediatric HIV care, including transmission, treatment, and testing protocols.
- Attitudes: Reflect participants’ personal beliefs, opinions, or perceptions about aspects of paediatric HIV care, such as disclosure, workload, and psychosocial support.
- Practices: Refer to self-reported behaviours or actions routinely taken in clinical settings related to paediatric HIV management.

The knowledge section assessed factual understanding of paediatric HIV, including ART regimens, co-infection management, and VTP protocols. Knowledge was assessed based on the 2019 VTP and ART Guidelines.^20^ Adequate knowledge was defined as achieving ≥ 50% correct responses. Attitudes were evaluated using questions exploring participants’ beliefs and perceptions toward HIV disclosure, psychosocial support, and responsibility for ART initiation. Practices captured self-reported clinical behaviours, such as use of gloves during blood exposure procedures, HIV counselling practices, and post-exposure protocols. Attitudes and practices were evaluated using a combination of questions from a prior KAP study^16^ and researcher-generated items, without the use of rating scales. Questionnaires were completed during participants’ free time and collected after shifts or lectures. Data were securely stored and managed using REDCap at a public university in the Free State.^21,22^

### Data analysis

Data analysis was performed by a trained biostatistician. Categorical variables were summarised using percentages, while numerical variables were summarised using mean for normally distributed variables and interquartile range (IQR) for skewed numerical variables. Pearson’s Chi-squared and Fisher’s tests were used for the analysis. The statistical significance for all tests was set at *P* < 0.05.

### Ethical considerations

Ethical clearance to conduct this study was obtained from the HSREC (reference number: UFS-HSD2002/0344/2607).

## Results

A total of 100 questionnaires were distributed during the study period. In total, 94 questionnaires were returned. Of these, 32 were from medical doctors and 62 were from nursing personnel. The median number of years worked by the nursing personnel group was 7 (IQR: 4, 16). Years of service were not summarised for the doctor group due to variability in training levels, which ranged from first-year interns to registrars at different stages of specialisation.

Table 1 depicts the demographic data of the participants. Table 2 provides a comparative summary of selected knowledge-based responses from nurses and doctors. Doctors consistently performed better on key items, including identification of the correct first-line ART regimen for children weighing 20 kg – 35 kg, understanding of dolutegravir (DTG), and the recommended approach to HIV and tuberculosis co-infection. Statistically significant differences were observed in several areas, reflecting a stronger overall knowledge base among doctors in paediatric HIV management.

**TABLE 1:** Socio-demographic data of doctors and nurses.

Variable	*N*	*n*	%
**Profession**	94	-	-
Nursing personnel	-	62	66.0
Medical doctor	-	32	34.0
**Level of education**	90	-	-
Matric	-	13	14.4
Diploma	-	35	38.9
Degree	-	42	46.7
**Rank of nursing personnel**	62	-	-
Enrolled nurse (EN)	-	21	33.9
Registered nurse (RN)	-	41	66.1
**Unit in which nursing personnel are currently working**	61	-	-
General paediatric ward	-	29	47.5
Paediatric intensive care unit	-	11	18.0
Neonatal high care unit	-	20	32.8
Kangaroo mother care/post-natal ward	-	1	1.6
**Rank of medical doctor**	32	-	-
First-year medical intern	-	11	34.4
Paediatric registrar	-	21	65.6

Note: Nursing personnel (*N* = 62), number of years in current post (median: 7, interquartile range: 4–16).

**TABLE 2:** Comparative analysis of HIV-related knowledge items between nurses and doctors.

Knowledge	*N*	Overall		Nurses		Doctors		*P* †
		*n*	%	*n*	%	*n*	%	
**Training in paediatric HIV management in the workplace**	88	-	-	-	-	-	-	0.022
Yes	-	28	31.8	13	23.2	15	46.9	-
No	-	60	68.2	43	76.8	17	53.1	-
**Most common route of transmission of HIV to a child, infant or neonate**	93	-	-	-	-	-	-	> 0.900
Mother to child	-	92	98.9	60	98.4	32	100	-
Sexual abuse	-	1	1.1	1	1.6			-
Sharing sharp objects, for example, needles, with an HIV-infected person	-	-	-	-	-	-	-	-
Not sure	-	-	-	-	-	-	-	-
**Regarding preventive treatment (VTP) of infants born to HIV-infected mothers who are on ART with a suppressed viral load and choose to breastfeed, which of the following is applicable?**	90	-	-	-	-	-	-	< 0.001
NVP during entire breastfeeding period	-	31	34.4	25	43.1	6	18.8	-
NVP for 6 weeks and AZT for 12 weeks	-	14	15.6	14	24.1	0	0.0	-
NVP only for 6 weeks	-	40	44.4	14	24.1	26	81.2	-
Not sure	-	5	5.6	5	8.6	0	0.0	-
**In a mother who is HIV-infected, with a viral load of more than 1000, which of the following regarding prophylaxis is correct?**	89	-	-	-	-	-	-	< 0.001
NVP only for 6 weeks	-	11	12.4	11	19.0	0	0.0	-
NVP and AZT for 6 weeks	-	38	42.7	20	34.5	18	58.1	-
The regimen will depend on the mother’s feeding choice	-	25	28.1	13	22.4	12	38.7	-
Not sure	-	15	16.9	14	24.1	1	3.2	-
**Regarding administration of co-trimoxazole (Bactrim), in the HIV-exposed infant, which is the most correct statement?**	91	-	-	-	-	-	-	0.008
Start it from 6 weeks onwards in both the low and high-risk infant	-	46	50.5	25	42.4	21	65.6	-
Start when the CD4 count is less than 25%	-	12	13.2	8	13.6	4	12.5	-
Continue until 5 years of age and stop thereafter only if CD4 criteria are met	-	4	4.4	1	1.71	3	9.4	-
Not sure	-	29	31.9	25	42.4	4	12.5	-
**What is the current recommended first-line ART regimen in an HIV-infected child who weighs between 3 and 20 kg?**	93	-	-	-	-	-	-	0.100
AZT + 3TC + NVP	-	24	25.8	20	32.8	4	12.5	-
ABC + 3TC + Kaletra	-	58	62.4	34	55.7	24	75.0	-
ABC + 3TC + EFV	-	-	-	-	-	-	-	-
Not sure	-	11	11.8	7	11.5	4	12.5	-
**What is the recommended first-line ART regimen in an HIV-infected child who weighs between 20 kg and 35 kg?**	91	-	-	-	-	-	-	< 0.001
ABC + 3TC + Kaletra	-	18	19.8	14	23.7	4	12.5	-
ABC + 3TC + DTG	-	22	24.2	3	5.1	19	59.4	-
TDF + 3TC + EFV	-	14	15.4	11	18.6	3	9.4	-
Not sure	-	37	40.7	31	52.5	6	18.8	-
**With regard to the management of HIV and tuberculosis co-infection, which statement is true?**	91	-	-	-	-	-	-	< 0.001
Complete 2 weeks of tuberculosis treatment and then initiate ART	-	62	68.1	31	52.5	31	96.9	-
Tuberculosis treatment and ART should be initiated jointly	-	2	2.2	12	20.3	0	0.0	-
ART should only be initiated after successfully completing tuberculosis treatment	-	11	12.0	8	13.6	1	3.1	-
Not sure	-	12	13.0	8	13.6	0	0.0	-
**Regarding HIV testing of the HIV-exposed neonate at birth**	92	-	-	-	-	-	-	< 0.001
HIV PCR for the neonate	-	67	72.8	35	58.3	32	100	-
HIV ELISA for the neonate	-	2	2.2	2	3.3	0	0.0	-
Not required if mother is virally suppressed and opting for formula. Can be done at 6 weeks	-	11	12.0	11	18.3	0	0.0	-
Not sure	-	12	13.0	12	20.0	0	0.0	-
**Regarding testing in the HIV-exposed infant/child**	87	-	-	-	-	-	-	< 0.001
The ELISA is the test of choice if the patient is older than 18 months	-	33	37.9	11	20.0	22	68.8	-
The PCR is the test of choice if the patient is older than 18 months.	-	6	6.9	6	10.9	0	0.0	-
After 18 months of age, both ELISA and PCR tests are acceptable.	-	39	44.8	29	52.7	10	31.2	-
Not sure	-	9	10.3	9	16.4	0	0.0	-
**Select the most correct statement regarding testing for HIV in children**	88	-	-	-	-	-	-	0.700
All sick infants/children should be tested on admission, even if recently tested negative.	-	58	65.9	36	64.3	22	68.8	-
All HIV exposed children should be tested at 6 weeks of age and then again at 10 weeks of age	-	17	19.3	10	17.9	7	21.9	-
The HIV test should be performed 4 weeks after stopping breastfeeding	-	3	3.4	3	5.4	0	0.0	-
Not sure	-	10	11.4	7	12.5	3	9.4	-
**When should the viral load test be done after initiation of ART?**	90	-	-	-	-	-	-	0.002
At diagnosis of HIV	-	22	24.4	19	32.8	3	9.4	-
After 6 months on treatment	-	53	58.9	26	44.8	27	84.4	-
Before changing to second-line regimen	-	6	6.7	6	10.3	0	0.0	-
Not sure	-	9	10.0	7	12.1	2	6.2	-
**Have you heard about the drug DTG, as part of HIV paediatric management?**	88	-	-	-	-	-	-	< 0.001
Yes	-	40	45.5	14	24.6	26	83.9	-
No	-	48	54.5	43	75.4	5	16.1	-
**The latest national clinical guidelines for the management of HIV in children, infants and neonates are from which year?**	94	-	-	-	-	-	-	0.001
2017	-	48	51.1	40	64.5	8	25.0	-
2019	-	32	34.0	15	24.2	17	53.1	-
2021	-	14	14.9	7	11.3	7	21.9	-
Not sure	-	-	-	-	-	-	-	-
**Knowledgeable**	94	-	-	-	-	-	-	< 0.001
Failed	-	52	55.3	48	77.4	4	12.5	-
Passed	-	42	44.7	14	22.6	28	87.5	-

Note: At the time of the survey, the terminology of PMTCT was still in use, but this is now being referred to as VTP (vertical transmission prevention).

ABC, Abacavir; ART, Antiretroviral therapy; AZT, Zidovudine; TDF, Tenoforvir; DTG, Dolutegravir; EFV, Efavirenz; NVP, Nevirapine; PCR, Polymerase Chain Reaction; ELISA, Enzyme-linked Immunosorbent Assay; 3TC, Lamivudine; PMTCT, prevention of mother-to-child transmission.

† , Pearson’s Chi-squared test; Fisher’s exact test.

### Knowledge

There were 14 questions in the knowledge section. The first question was for participants to indicate whether or not they had previously received training and was therefore not a scored question. Of the total study participants, only 44.7% were assessed as having adequate knowledge. Most of the nursing personnel (77.4%) failed the knowledge section of the questionnaire. Among nursing personnel (of whom 34% were enrolled nurses and 66% were professional nurses), key areas of limited knowledge included: prophylaxis for HIV-exposed infants, recommended first-line antiretroviral regimens, HIV testing protocols across different paediatric age groups, awareness of new antiretroviral agents introduced in the latest guidelines, and the management of HIV in the context of co-infections, particularly tuberculosis. The difference between the knowledge of enrolled nurses and professional nurses was found to be statistically significant (*P* = 0.001).

Only 12.5% of the medical doctors failed the knowledge section. A comparison of knowledge between interns and registrars in this study showed registrars to be superior in knowledge than interns. Even though the number of interns in this study was small, the study found gaps in knowledge in the following areas: first-line treatment regimens and new drugs in treatment guidelines.

The difference in performance in terms of knowledge between the doctors and the nurses was statistically significant (*P* < 0.001).

**FIGURE 1:**
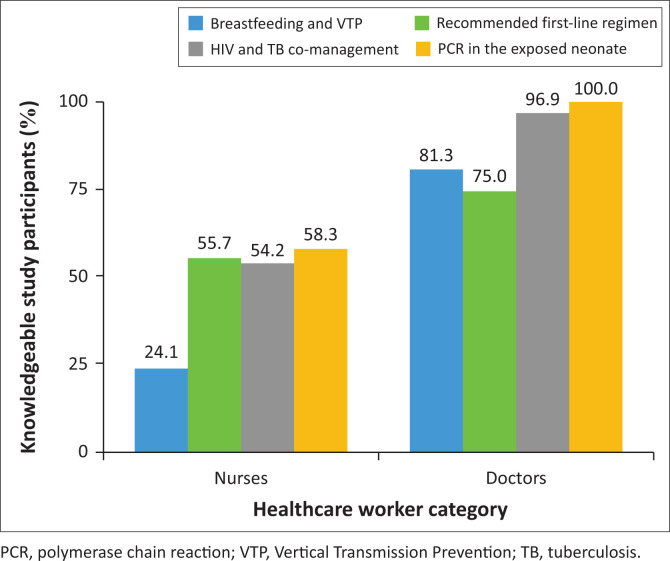
Proportion of knowledgeable healthcare workers.

Responses in the knowledge section, for which there was a statistically significant difference, are displayed in Figure 1. The graph depicts the proportion of the study participants that were knowledgeable on the particular subject matter.

Knowledge levels varied considerably between nurses and doctors across all assessed domains. Doctors demonstrated consistently higher knowledge across all four topics. The greatest disparity was observed in the domain of breastfeeding and vertical transmission prevention (VTP), where only 24.1% of nurses were knowledgeable compared to 81.3% of doctors. Similarly, for HIV and tuberculosis co-management, 96.9% of doctors were knowledgeable, in contrast to 54.2% of nurses. In the domain of PCR testing in the exposed neonate, 100% of doctors and 58.3% of nurses were knowledgeable. The subject matter with the least disparity was the recommended first-line regimen, with 75.0% of doctors and 55.7% of nurses demonstrating adequate knowledge.

#### Knowledge on treatment regimen

Half of the participants (50.5%) agreed with commencing Co-trimoxazole from 6 weeks onwards. Almost a third (62.4%) of the participants knew the recommended first-line regimen for a patient with a weight between 3 kg and 20 kg. Only 24.2% of the participants opted for a DTG-based first-line regimen for patients in the weight category of 20 kg – 35 kg. Regarding DTG, the majority of the doctors (83.9%) knew of it whereas only 24.6% of the nursing personnel knew of it. This was statistically significant (*P* < 0.001).

#### Knowledge on HIV testing and viral load measurement

Almost a third (65.9%) of all study participants indicated that all infants should be tested for HIV on admission, even if they had recently tested negative. Regarding when viral load testing should be done, 58.9% of all study participants indicated that it should be done 6 months after treatment initiation. Over half of all study participants (51.1%) indicated that the latest HIV treatment and VTP guidelines were from 2017. It is important to note that this may reflect uncertainty about the publication date rather than an actual lack of awareness of updated clinical content.

### Attitudes

**FIGURE 2:**
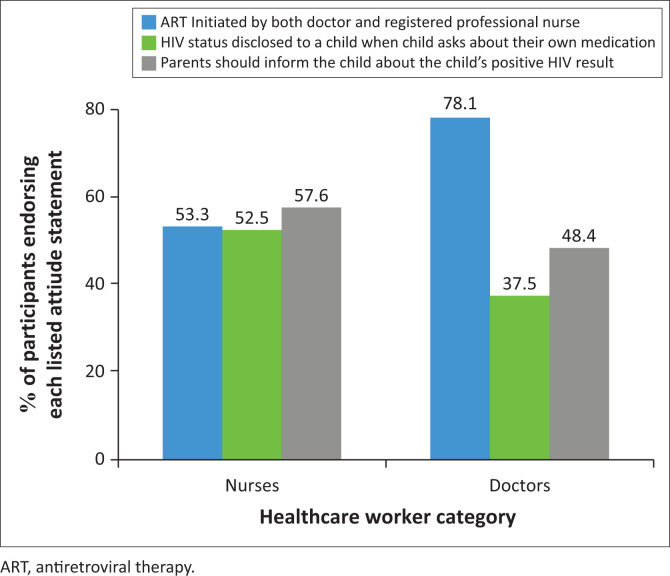
Proportion of nurses and doctors responding ‘yes’ or selecting key attitude-related options.

The majority of the participants (62%) were of the opinion that ART in the paediatric population can be initiated by both doctors and nurses. Almost half (47.3%) of the total participants indicated that HIV infection disclosure should be done when the child asks about their treatment and did not link it to any age. Although the HCW do the HIV testing and counselling, over half (54.4%) of the participants indicated that it is the parents who should disclose the HIV status to their children. The difference in opinions between the two respective HCW groups is represented in Figure 2. The opinions on HIV-infected patients expressed by HCW are depicted in Table 3.

**TABLE 3:** Proportion of nurses and doctors agreeing with selected statements about HIV-infected children.

Description	%	
	Nurses	Doctors
HIV-infected children need counselling	100.0	100.0
HIV-infected children need education about HIV	100.0	100.0
HIV-infected children face discrimination from other children	84.5	90.0
HIV-infected children create an extra workload	70.0	29.0
Caring for HIV-infected children can negatively affect the mental and physical wellness of a healthcare worker	15.3	38.7

#### Training

**FIGURE 3:**
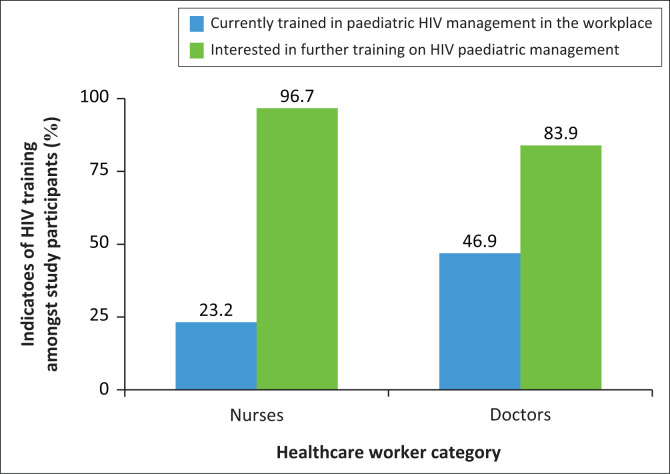
Indicators of training in HIV.

Insufficient training on HIV in the workplace was expressed by 61.8% of HCW. This correlates with 92.3% of participants indicating an interest in further HIV training, as indicated in Figure 3.

### Practices

**FIGURE 4:**
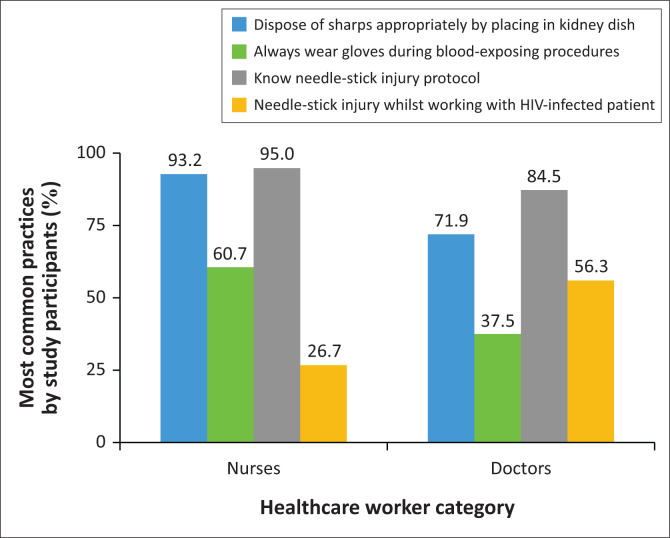
Factors associated with blood exposing procedures.

Approximately 59.2% participants indicated that they always wore gloves when working with HIV-infected patients. 56.2% of doctors did not always wear gloves. Less than 5% of total HCW never wore gloves. The majority of participants (94.6%) indicated that there was a higher chance of wearing gloves if they knew the patient was HIV infected. The important factors associated with blood-exposing activities as part of daily practice are depicted in Figure 4.

Other aspects of practices were related to counselling. The majority of both nurses (53.3%) and doctors (78.1%) indicated that they would continue counselling the parents until they consented to an HIV test of their children if they refused initially. Almost 70% of the participants had at least disclosed a positive HIV status to a patient once before. Over half (54.5%) of the participants indicated they provide counselling to HIV-infected mothers about breastfeeding. More than two-thirds of study participants (71.4%) provided ongoing counselling to mothers of HIV-infected children in general. Only 57.4% of doctors and 60% of nurses indicated that they actively do research on paediatric HIV. When admitting an HIV-exposed neonate of a mother with an unknown viral load, only 67.5% of HCW indicated that they actively sought the maternal viral load.

## Discussion

This study evaluated the KAP of HCWs involved in paediatric and neonatal HIV care. Key findings included significantly lower knowledge scores among nurses compared to doctors, particularly regarding ART regimens, co-infection management, and infant prophylaxis. Despite these gaps, attitudes were overwhelmingly positive across both groups, with unanimous support for HIV education and counselling for HIV-infected children. Reported practices were mostly appropriate, although some variation was noted in areas such as disclosure procedures and post-exposure precautions. Importantly, most participants indicated a strong interest in further training, highlighting an opportunity to strengthen HIV service delivery through targeted educational interventions.

This study further demonstrated that, in comparison to medical doctors, nursing personnel had more gaps in HIV-related knowledge. These two professional groups differ in the level and type of training they receive, which may partially explain the observed difference. However, it is important to note that not all nurses are NIMART-trained or expected to manage HIV independently. The inclusion of nurses with varied roles and training levels may have contributed to the variation in knowledge observed, and this should be taken into account when interpreting the findings.

The findings of this study align with those from a Cameroonian study conducted across 12 health facilities, which found that while healthcare providers demonstrated adequate knowledge in some aspects of paediatric HIV care, significant gaps remained – particularly in ART initiation and comprehensive management.^16^ Similarly, our study identified knowledge deficiencies among nursing personnel, likely reflecting variations in scope of practice and training levels, including the absence of NIMART training for some participants.

Inadequate training has consistently been identified as a key contributing factor to reported low levels of HIV-related knowledge among nursing personnel.^23,24,25^ Although this study did not investigate the underlying reasons for inadequate training, previous research suggests that structural and systemic challenges – rather than individual neglect – are often responsible.^23,24,25^ Other reported barriers to paediatric HIV care, such as institutional limitations, lack of standardised tools, and caregiver-related challenges, were not within the scope of this study. Future research could explore these broader factors in our setting to provide a more comprehensive understanding of the challenges faced by HCWs.^23,24,25^

There may be other factors contributing to the low levels of knowledge observed among nursing personnel in this study, aside from the amount of previous HIV training. One factor to consider is the role of the nurse. In this setting, nursing personnel are generally more involved in administering medications rather than making treatment decisions. This is different from primary healthcare settings where programmes like NIMART are operational and are valuable in empowering nurses with knowledge of HIV management and treatment decisions. Another factor to consider is the exposure to new drugs in the treatment guidelines. For example, regarding the novel drug DTG, it is possible that, at the time of this data collection (July to September 2022), only a limited number of patients in the wards were on the drug. This may explain the poor performance of nursing personnel on questions related to treatment guidelines, as their exposure to such drugs was limited.

To the researchers’ knowledge, there are no studies documenting the KAP of doctors caring for HIV-infected paediatric patients in South Africa in over a decade.^26^ The difference in levels of knowledge between interns and registrars is expected and is reflective of the difference in terms of the level of medical training and clinical experience gained over the years. More important than comparing the two groups was identifying gaps in knowledge in the intern group. This is important as interns soon become community service medical officers who may be expected to manage both complex and non-complex paediatric HIV-related cases. Of note, this group of interns had not yet rotated through the Family Medicine rotation of their training, which is another rotation that may further expose them to HIV in paediatrics.

The second and third focus areas of the study were to establish what the attitudes and practices of HCW caring for HIV-infected paediatric patients were. Although there are objective ways to ascertain whether attitudes are positive or negative, for example by making use of a Likert scale, the attitudes and practice section of this study rather explored the thinking processes of HCW around issues pertaining to daily paediatric HIV care.

There were variations in the attitudes and reported practices between medical doctors and nursing personnel in this study. While no formal correlation analysis was conducted between KAPs, certain trends appeared to differ by professional category. For example, a higher proportion of nurses reported routine glove use during procedures involving potential blood exposure. This contrasts with doctors, who reported glove use less consistently despite likely performing a greater number of invasive procedures in this setting.

However, it is important to interpret this finding cautiously, as procedural exposure alone may not fully explain the discrepancy. Factors such as role-specific training, perceived risk, or access to protective equipment may also contribute. Moreover, all HCWs – including those not directly involved in procedures – are at risk of occupational exposure through events such as resuscitations or improper sharps disposal. These findings suggest a need to reinforce standard infection-prevention practices across all HCW categories.

While some studies have reported a higher incidence of needle-stick injuries among nursing personnel,^27,28^ the findings of this study differed. This discrepancy may reflect differences in clinical settings, patient populations, or the specific roles and responsibilities of HCWs in paediatric care. Additionally, variations in adherence to infection prevention measures, including glove use, may influence perceived or actual exposure risks. However, it is important to note that glove use offers protection but does not eliminate the risk of needle-stick injuries.

One practice in which both study participant groups were equal was that of their response to the unknown maternal HIV viral load. The importance of this lies in the mitigation of the side effect profile of prophylactic ART on the neonate as well as increasing the risk of transmission, by using only nevirapine (NVP) instead of combining it with zidovudine (AZT) in cases where the maternal HIV viral load is unknown. The dose-dependent side-effect profile of AZT on neonates has been well described.^29^

There was a common thread that seemed to exist between attitudes and practices relating to HIV counselling, testing and disclosure in this study. HIV status disclosure to children remains contentious and is certainly one area that has received little attention in literature and probably less so in clinical practice.^30^ From this study, this was demonstrated by attitudes of uncertainty regarding the process and its associated legal implications. The *Children’s Act 38 of 2005* is an excellent resource that provides guidance on the legalities surrounding the HIV testing process in children.^31^

Although there are recommendations and guidelines on when and how to disclose to children, there is no evidence to suggest which one way is superior.^32,33^ It is well documented in literature that many HCW do not feel equipped to disclose to children as they are not adequately trained for this process.^23,24,25^ This was also evident in this study. Ideally, this should not be the case, as South Africa provides specific guidelines from the National Department of Health with an outlined stepwise approach on the disclosure process to children.^33^

A positive three-way correlation between KAP of HCW suggests that improvement in any one of these factors may positively influence the other two.^19^ Evidence indicates that HCWs with over 5 years of experience in a specific medical field and who have received training in that area tend to demonstrate better knowledge and more favourable attitudes.^34^ Although our nursing personnel participants have more than 5 years of experience, the generally low levels of reported training likely contributed to the lower knowledge levels observed in this study. Since information on NIMART training status was not collected, it is not possible to determine its influence directly; however, it is plausible that the absence of such specialised training partially explains these findings. Nevertheless, it is encouraging that participants expressed a positive attitude towards improving their knowledge, indicating readiness for further education and capacity building.

### Strengths and limitations

Participation was voluntary and, as a result, some questionnaires were not returned. Of the 100 questionnaires distributed, 94 were returned, translating to a response rate of 94%. Of those that were returned, not all questions were answered. This exposes the results to potential bias. This study was conducted on a small sample of doctors and nurses working at Pelonomi Tertiary Hospital and therefore the results cannot be generalised to all paediatric and neonatal units in South Africa, as this was an explorative study whose findings are based on focus group data from a small number of HCWs. As a result, a causal relationship is not necessarily implied by the associations found.

Consultant paediatricians were not included among the study participants. Their exclusion is a notable limitation, as their insights – given their role in overseeing the comprehensive management of paediatric and neonatal patients – would have added valuable perspective. The data from the intern group are also not representative, because of the low number of interns involved in the study during data collection. Self-reported attitudes and practices of participants may overestimate, or underestimate, the positive HCW practices and attitudes. The researchers acknowledge that not using formal rating scales may limit the precision and standardisation of attitude and practice measurements.

Regarding the questionnaire, while it assessed whether or not healthcare workers had ever disclosed a positive HIV test result, it did not clarify whether this referred specifically to disclosure to a child. Lastly, for the nurses who had received prior HIV training, the questionnaire did not explore whether or not this was specifically in NIMART.

### Implications or recommendations

The recommendations from this study are as follows:

- Provide feedback to the two respective professional groups about the findings of the study.
- Advocate for interactive training sessions on paediatric HIV in order to address the gaps in knowledge identified.
- Create separate training platforms specifically geared at HIV counselling and disclosure to the paediatric patient by all HCW.
- Encourage HCW to be more proactive about their response to an unknown maternal HIV viral load.

## Conclusion

This study revealed generally low levels of HIV-related knowledge among nursing personnel across different training levels, while attitudes were favourable and optimistic. Practices were largely appropriate and acceptable. The findings underscore a clear need for enhanced HIV-related training for HCWs in the paediatric and neonatal units at Pelonomi Tertiary Hospital. Importantly, the positive attitudes suggest readiness for implementing such training initiatives.

These results have implications beyond a single institution. They reflect challenges that are likely shared across similar public healthcare facilities in South Africa, particularly those with a high HIV burden. Strengthening paediatric HIV training nationally – especially among nursing personnel – could contribute to improved quality of care and help sustain the gains made in reducing vertical transmission.
